# Dermoscopy of skin metastases from breast cancer: two case reports

**DOI:** 10.1186/s13256-018-1803-z

**Published:** 2018-09-22

**Authors:** Awatef Kelati, Salim Gallouj

**Affiliations:** Department of Dermatology, University Hospital of Fez, Fez, Morocco

**Keywords:** Skin metastases from breast cancer on mastectomy scar, Dermoscopy

## Abstract

**Background:**

Cutaneous metastatic breast cancer is the most common cutaneous metastatic malignancy in women. The assessment of cutaneous metastatic disease can be perplexing because the clinical presentation appears similar to other skin malignancies like angiosarcoma or melanoma, or benign diseases like cellulitis and lymphedema. To date, only a limited number of dermoscopic images of cutaneous metastatic solid tumors, especially breast cancer, have been published.

**Case presentation:**

The authors report two Moroccan cases highlighting dermoscopy as a quick tool to recognize skin metastasis of breast cancer in two different clinical presentations. A 51-year-old Moroccan woman presented with nodules of various sizes on and around a mastectomy scar, and a 65-year-old Moroccan woman presented with cellulitis-like lesions on her chest wall and her back. Dermoscopic features were similar in the two cases with findings of yellow central areas, polymorphic vessels, whitish bright lines, whitish structureless areas, and linear irregular fissure-like depressions on a pink-orange background.

**Conclusions:**

The recognition of dermoscopic patterns of cutaneous metastasis of breast cancer is not only useful to facilitate diagnosis at an early stage and to rule out other differentials, especially in difficult presentations such as cellulitis-like lesions or lymphedema, but it may also be used by physicians in monitoring mastectomy scars.

## Background

Metastatic cutaneous lesions are seen more commonly in breast cancer than in any other malignancy in women, exceeding 20% of all cutaneous metastases [1]. The presence of skin metastases signifies widespread systemic disease and a poor prognosis [2]. Patients present with a variety of symptoms ranging from non-painful, single or multiple, hard, firm, indurated skin to tiny seed-like solid papules and large egg-sized lesions, with sometimes an edema of the skin of the breast, known as the orange peel sign, without any specific clinical diagnostic criteria. The chest wall, the abdomen, the back, and the upper extremities are common sites [3].

Assessment of cutaneous metastatic disease after mastectomy can be perplexing because the clinical presentation appears similar to other skin diseases such as cellulitis or lymphedema [1]. Although dermoscopy may provide a useful method for the differentiation between the diagnosis of metastasis to the skin and non-neoplastic dermatological diseases or other malignancies, the dermoscopic patterns of breast cancer metastases have not been well described.

We report two cases of breast cancer metastases with an emphasis on the dermoscopic patterns of the disease.

## Case presentation

The authors report two Moroccan cases of dermoscopy in skin metastasis of breast cancer with two different clinical presentations; the dermoscopic examination was performed using a Dermatoscope Delta® 20 (Heine; Herrsching, Germany) with polarized light and without immersion.

Case 1 was a 51-year-old Moroccan woman diagnosed as having infiltrating ductal carcinoma of the left breast. Case 2 was a 65-year-old Moroccan woman diagnosed as having infiltrating ductal carcinoma of the right breast. They underwent mastectomy and axillary node dissection followed with adjuvant hormone and chemotherapy. After a remission period of 14 months (Case 1) and 10 months (Case 2), they were referred to our hospital for painful lesions on the surface of their trunk, chest, and back.

**Fig. 1:**
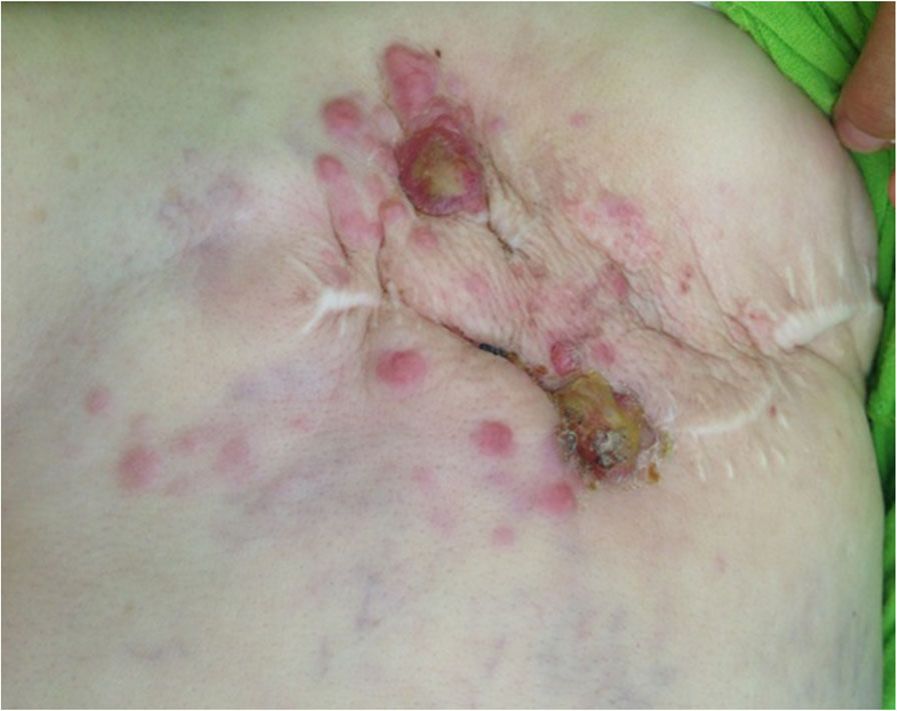
Case 1: Clinical presentation. Multiple irregularly distributed pink nodules around the indurated mastectomy scar on the left chest wall

**Fig. 2:**
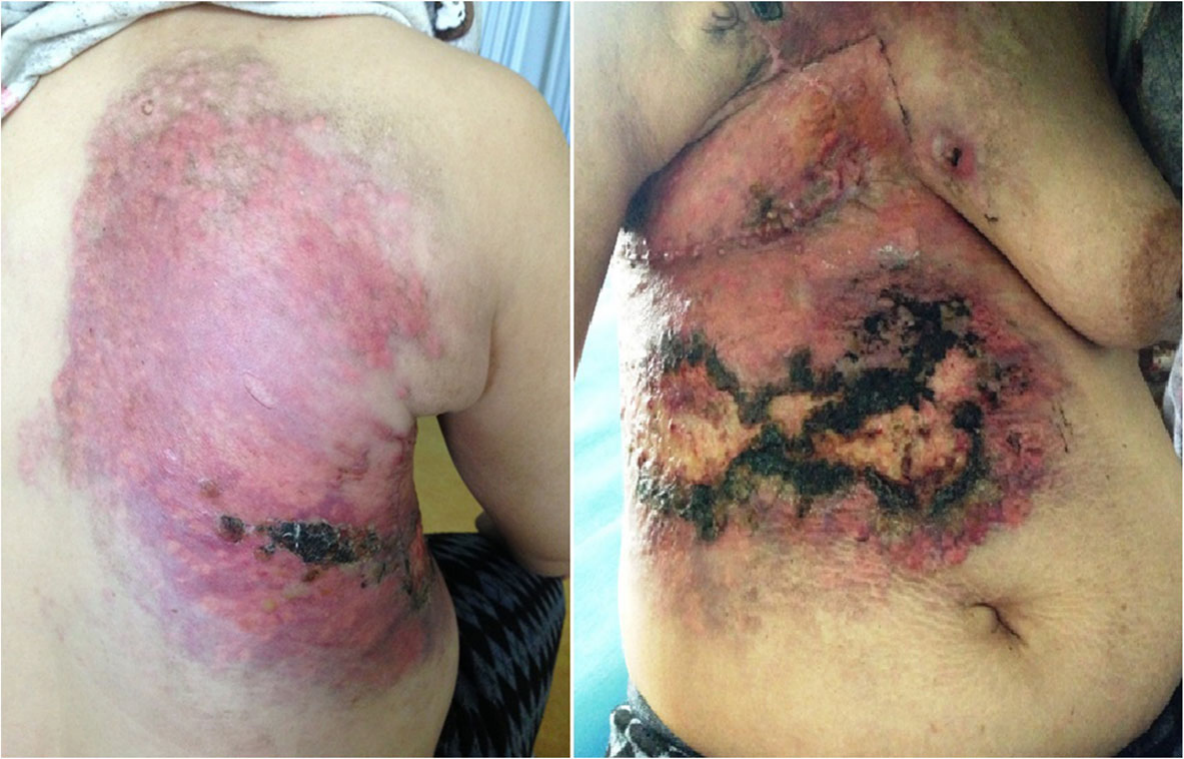
Case 2: Clinical presentation. Diffusely swollen, erythematous, indurated, and ulcerated skin with a cellulitis-like appearance of the right chest wall and the back

**Fig. 3:**
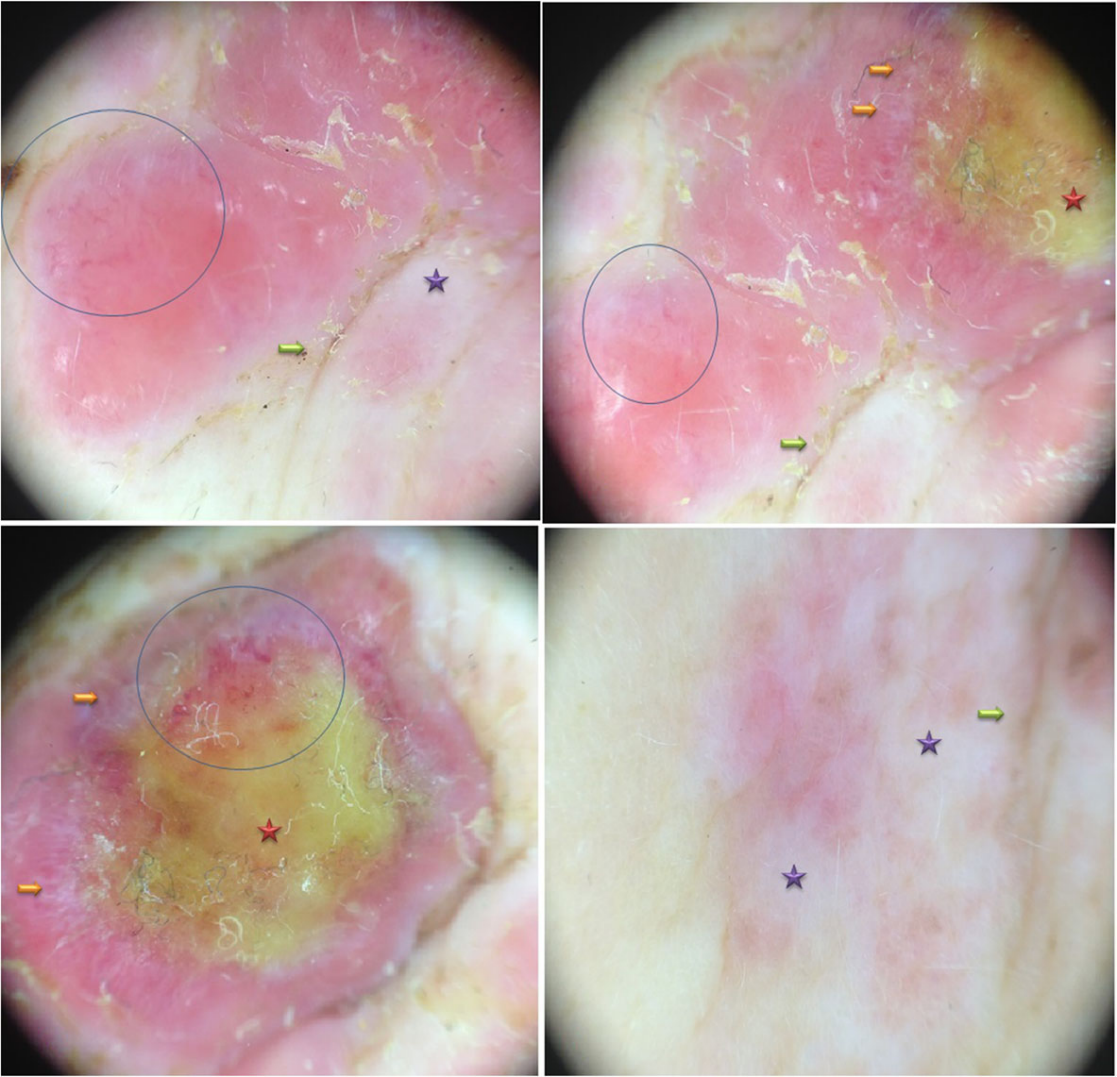
Case 1: Dermoscopy of the skin metastases. Linear, irregular, arborizing and polymorphic vessels at the periphery of purples lesions (*blue circle*), structureless yellow areas (*red star*), bright white lines at the periphery of yellow areas (*orange arrow*), white structureless areas (*violet star*), linear skin depressions as fissure-like structures (*green arrow*)

**Fig. 4:**
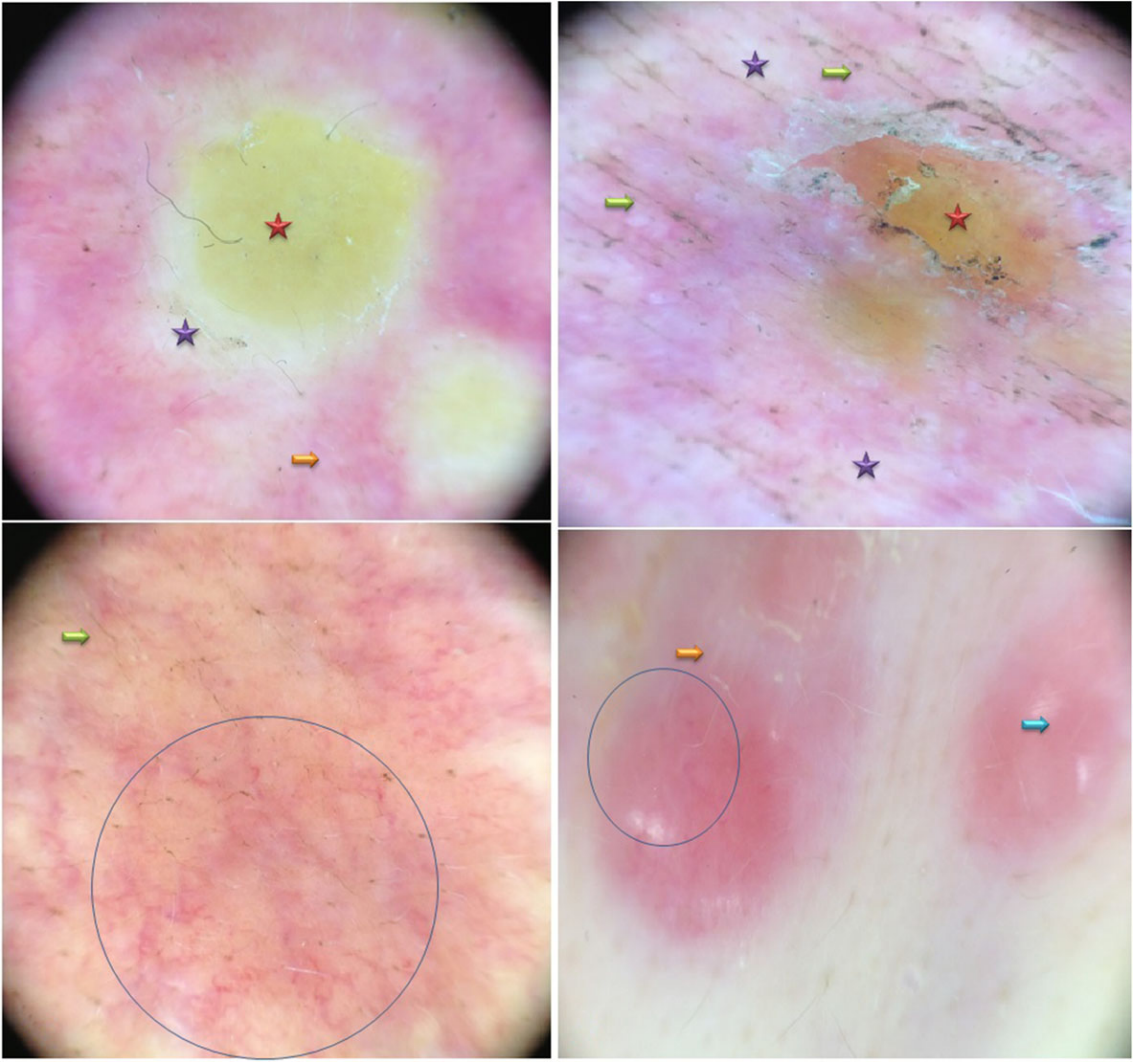
Case 2: Dermoscopic image of the skin metastases. Linear, irregular, arborizing and polymorphic vessels (*blue circle*), structureless yellow or yellow-orange areas (*red star*), white lines (*orange arrow*), white structureless areas or around yellow structures (*violet star*), linear skin depressions as fissure-like structures (*green arrow*), and pink structureless areas (*blue arrow*)

For Case 1, a physical examination revealed irregularly distributed pink nodules of various sizes with a large firm, indurated skin on and around the mastectomy scar of her left chest (Fig. 1). For Case 2, a physical examination revealed a diffuse well-demarcated erythema and edematous cellulitis-like skin on the right side of her chest wall and her back, with a central ulceration on her abdominal wall (Fig. 2) and palpable lymphadenopathy in her bilateral anterior cervical and supraclavicular chains. Dermoscopic examination of the two cases revealed a pink-orange background, yellow central areas, linear irregular and polymorphic vessels, whitish bright lines, whitish structureless areas, and linear irregular fissure-like depressions. A recurrence of ductal carcinoma was confirmed with skin biopsies, and the patients were referred to the oncology department for further investigations and appropriate management (Figs. 3 and 4).

## Discussion

All types of cancer may metastasize to the skin, with the frequency of occurrence ranging from 0.2 to 9% among autopsies carried out on patients with cancer. Skin metastases may occur synchronously or metachronously with the diagnosis of the primary tumor. Occasionally, skin metastases may represent an initial manifestation of an occult internal carcinoma. Breast and lung cancer are the most common primary types of cancer that metastasize to the skin [4].

Patients with breast cancer require differentiation between skin metastasis and benign dermatological disease. Differences between cutaneous metastases and cellulitis or lymphedema were found most definitively on the histologic study of tissue biopsy [5].

Advanced metastatic breast cancer is difficult to cure, and an eventual resistance to cytotoxic treatment is expected, progression of cutaneous metastases may lead to a fungating mass that would require skin and wound management [5]. Fungating wounds can decrease quality of life by negatively impacting psychological well-being and increasing social isolation [6]. This is why the recognition of cutaneous metastasis at an early stage is very important for the therapeutic approach, because surgery of limited lesions may be performed, which is not possible for advanced stages. For this reason, we think that dermoscopy may be of great help in recognizing these types of cutaneous metastasis.

To date, only a limited number of dermoscopic images of cutaneous metastatic solid tumors have been published [4]. Cutaneous metastatic breast cancer dermoscopy was recently described in two papers of three case reports; the first case reported nodular hyperpigmented metastatic breast cancer, with features of peripheral globules and blue–white veil mimicking a melanoma [7]; recently, in two other cases, findings of polymorphous vascular structures, whitish depigmentation, umbilicated pits with a tendency for forming linear fissure-like structures with small, lateral depressions were reported. These last features were also noticed in our two cases. As a result, a characterization of metastatic breast cancer may be performed based on these patterns; yellow areas were an additional feature in our cases.

Polymorphous and atypical vessels are the most frequent vascular structures characterizing malignancy [8]; they were also observed in these cases of cutaneous breast metastasis. However, these structures may be misdiagnosed as an angiosarcoma or a lymphangiosarcoma in Stewart–Treves syndrome after mastectomy where we can also find white lines as another sign of malignancy [9], the other dermoscopic signs would be of great help, like the yellow-orange color and the fissure-like depressions that were described in our two cases.

These polymorphic vessels may also have a prognostic value: the greater the density of the vessels, the more the disease is invasive, which was observed in our second case where the vascularization is abundant and it was concordant with an advanced clinical stage; this deserves further investigation in prospective studies.

## Conclusions

This is the second dermoscopic description of non-pigmented cutaneous metastasis of breast cancer in two different clinical presentations with similar dermoscopic features. This may facilitate the monitoring and the recognition of these tumors at an early stage by dermoscopic examination of the mastectomy scar before the stage of widespread nodules or cellulitis-like erythema.
